# Genetic Polymorphisms of Long Non-coding RNA Linc00312 Are Associated With Susceptibility and Predict Poor Survival of Nasopharyngeal Carcinoma

**DOI:** 10.3389/fcell.2021.698558

**Published:** 2021-07-16

**Authors:** Zhen Guo, Mei-Hua Bao, Yun-Xia Fan, Yan Zhang, Hai-Yan Liu, Xiao-Long Zhou, Ben Wu, Qing-Qing Lu, Bin-Sheng He, Xu-Ying Nan, Jiao-Yang Lu

**Affiliations:** ^1^Academician Workstation, Changsha Medical University, Changsha, China; ^2^Hunan Key Laboratory of the Research and Development of Novel Pharmaceutical Preparations, Changsha Medical University, Changsha, China; ^3^Geneis Beijing Co., Ltd., Beijing, China; ^4^School of Chemistry and Chemical Engineering, Hainan Normal University, Haikou, China

**Keywords:** nasopharyngeal carcinoma, linc00312, polymorphism, susceptibility, survival

## Abstract

**Background:**

Linc00312 is dysregulated in nasopharyngeal carcinoma (NPC) and participates in the initiation and progression of NPC. Our previous studies suggested that linc00312 was able to enhance the sensitivity of NPC cells to irradiation and NPC patients with higher expression of linc00312 was associated with better short-term curative effect and overall survival. The single nucleotide polymorphisms (SNPs) of lncRNAs may influence the disease course and outcome by affecting the expression, secondary structure or function of lncRNAs. However, the role of SNPs in linc00312 on the occurrence and survival of NPC remains unknown.

**Methods:**

We recruited 684 NPC patients and 823 healthy controls to evaluate the association between linc00312 SNPs and NPC susceptibility by using multivariate logistic regression analysis. Kaplan-Meier analysis and Cox proportional hazards regression were applied to assess the effect of linc00312 SNPs on the survival of NPC patients. The relative expression of linc00312 in NPC tissues was determined by real-time PCR. The interaction between linc00312 and mir-411-3p was explored by luciferase reporter assay. *In silico* prediction of the changes on linc00312 folding structure was conducted by RNAfold WebServer.

**Result:**

We demonstrated that rs12497104 (G > A) GA genotype carriers had a higher risk than others for suffering from NPC (GA *vs* GG, OR = 1.437, *P* = 0.003). Besides, patients with rs12497104 AA genotype showed a poorer overall survival in contrast to GG genotype (AA *vs* GG, HR = 2.117, *P* = 0.011). In addition, the heterozygous carriers of rs15734 (G > A) and rs164966 (A > G) were correlated with decreased risk of NPC (GA *vs* GG, OR = 0.778, *P* = 0.031; GA *vs* AA, OR = 0.781, *P* = 0.033, respectively). We found that the three SNPs might influence the expression of linc00312 in a genotype specific feature. The local centroid secondary structure as well as the minimum free energy of linc00312 were changed following the candidate SNPs alterations. Besides, we revealed that the G to A alteration at rs12497104 disrupted the binding between mir-411-3p and linc00312.

**Conclusion:**

Our results indicated genetic polymorphisms of linc00312 might serve as potential biomarkers for NPC carcinogenesis and prognosis.

## Introduction

Nasopharyngeal carcinoma has a remarkable geographic distribution and is relatively predominant in Southern China. According to the statistics report of nasopharyngeal carcinoma (NPC) in 2018, more than 70% of new cases are in east and southeast Asia, with an age-standardized rate (world) of 3.0/100,000 in China to 0.4/100,000 in white populations (Bray et al., 2018). The etiology of NPC is multifactorial and is widely suspected to be complex interactions of genetic predisposition, Epstein–Barr virus infection, and environmental factors (Paul et al., 2018).

Distinguished from other types of head and neck cancer, NPC is more aggressive due to extensive local infiltration, early lymphatic spread, and high tendency of hematogenous dissemination. More than 70% of patients are classified as locoregionally advanced stages at the time of diagnosis (Zhang et al., 2019b). Currently, radiotherapy and platinum-based chemotherapy are the mainstay treatment modality for NPC (Chen et al., 2019). The 3-years failure-free survival is around 80% for NPC patients receiving induction chemotherapy plus concurrent chemoradiotherapy (Sun et al., 2016; Zhang et al., 2019a).

It is now well recognized that genetic aberrations play a vital role in the pathogenesis, progression and prognosis of NPC (Lin et al., 2014; Tang et al., 2018). Previous studies have reported several susceptibility and recurrent associated loci of NPC, for example, *CYLD*, *TRAF3*, *MST1R*, *TNFRSF19*, *MECOM*, and *CDKN2A–CDKN2B* (Bei et al., 2010; Dai et al., 2016; Li et al., 2017). As we can see, most of the identified loci are located within coding regions. Noteworthy, results from GWAS showed only 7% of diseases associated loci was located in protein-coding regions, indicating genetic variations in non-coding regions are likely to exert important functions in disease (Freedman et al., 2011).

Long non-coding RNAs (lncRNAs) lack the capability of protein-coding and are once considered to be simply transcriptional “noise”. Recently, emerging evidence have shown that lncRNAs participates in the regulation of many cellular biological processes (Lin and Yang, 2018). Deregulation of lncRNAs are closely related to tumorigenesis and prognosis of cancers (Wu and Hann, 2018). Single-nucleotide polymorphism (SNP) is one of the most commonly occurred type of genetic variants within lncRNA genes. The SNPs may exert its effect by affecting the expression, secondary structure or function of lncRNAs, thus influencing the disease course and outcome (Minotti et al., 2018).

In the present study, we focus on linc00312, which is dysregulated in NPC. The expression of linc00312 in NPC tissues is negatively correlated with tumor size (Zhang et al., 2013). Linc00312 exerts dual role on NPC cells as it inhibits cells proliferation, but increases cell adhesion and invasion (Huang et al., 2009). Our previous study revealed that overexpression of linc00312 enhanced the sensitivity of NPC cells to irradiation through regulating DNA damage response. NPC patients with higher expression of linc00312 were associated with longer overall survival (Guo et al., 2021). All of these indicates linc00312 might serve as a potential biomarker for NPC.

To date, the functional significance of SNPs in linc00312 locus is still unclear. Given the fact mentioned above, we speculate that the functional genetic variations in linc00312 may affect its expression and/or function, which finally influence the carcinogenesis and prognosis of NPC. In order to verify the conjecture, we selected three SNPs with potential regulatory feature of linc00312 and performed a case-control study to explore the role of linc00312 variations on NPC risk and survival.

## Materials and Methods

### Study Population

684 histopathology-confirmed NPC patients were enrolled at Hunan Provincial Cancer Hospital between 2014 and 2015. Meanwhile, 823 cancer-free volunteers taking physical examination at Xiangya Hospital were enrolled as controls. Moreover, we collected 82 NPC tissue biopsies from patients taking nasopharyngoscopy inspection. The patients who did not receive IMRT radiation technique or abandoned treatment or were out of contact were excluded in the survival analysis. All of the patients underwent platinum-based chemoradiotherapy. A total of 68–74 Gy irradiation was administered to the primary tumor for 7 weeks. The chemotherapy regimens in the present study including: platinum + paclitaxel (TP); platinum + 5-fluorouracil (FP); platinum + docetaxel (DP); platinum + gemcitabine (GP); cisplatin alone (DDP); nedaplatin alone (NDP). This study was approved by the Independent Ethical Committee of Institute of Clinical Pharmacology, Central South University (CTXY-140007-2) and all the participants signed the informed consent at the time of enrollment.

### Candidate SNPs Selection

Three online bioinformatic databases, including ENCODE, ENSEMBL and lncRNASNP were applied to select potentially functional SNPs that located in miRNA binding site/3′-untranslated region (3′-UTR)/enhancer region/5′-untranslated region (5′-UTR)/alternative splice region/open chromatin region. Finally, we selected three SNPs of linc00312 [rs12497104 (G > A), rs15734 (G > A), rs164966 (A > G)] that have never been reported for further study.

### Genotyping

Genomic DNA was isolated from peripheral blood lymphocytes and NPC tissues using the QIAamp DNA Mini and Blood Mini Kit (Qiagen Inc., Valencia, CA, United States). The concentrations and purity of DNA were measured using NanoDrop^TM^ 1000 spectrophotometer. Genotypes of the candidate SNPs were determined by using Sequenom MassARRAY iPLEX (Sequenom, Inc., San Diego, CA, United States). The call rate threshold was at least 95%.

### Real-Time PCR

Total RNA was extracted from NPC tissues and reverse-transcribed to cDNA by using a PrimeScript^TM^ RT kit (Takara, Japan). LightCycler 480 system was utilized for amplification of cDNA using a SYBR Premix Ex Taq^TM^ kit (Takara, Japan). The relative expression of linc00312 was calculated by 2^–ΔΔ*Ct*^ method. The primers are as the following: *GAPDH* forward: 5′-ACAACTTTGGTATCGTGGAAGG-3′ and reverse: 5′-GCCATC ACGCCACAGTTTC-3′; *linc00312* forward: 5′-GATCTATG GCCCATCATTCTTT-3′ and reverse: 5′-GTCCATCATGTAGC AAGCAGT-3′.

### Dual Luciferase Reporter Assay

The human NPC cell lines HONE1 and HNE1 were cultured in RPMI 1640 medium (Invitrogen, Carlsbad, CA, United States) containing 10% FBS (Invitrogen, Carlsbad, CA, United States) and 1% penicillin-streptomycin (100 units/mL) at 37°C in a 5% CO_2_ incubator. The 3′-UTR of linc00312 covering wild-type of rs12497104 was directly synthesized by Genechem (Genechem, Shanghai, China) and cloned into *Xba*I site of pGL3 promoter vector to get the wild-type vector (WT). Site-directed mutagenesis at rs12497104 was utilized to get the mutant-type vector (MUT). DNA sequencing was performed in order to make sure of the sequence of these vectors. HONE1 and HNE1 cells were co-transfected with Renilla vector, WT\MUT reporter vector, mir-411-3p mimics\mimics control or mir-411-3p inhibitor\inhibitor control using Lipofectamine 3000 (Invitrogen, Carlsbad, CA, United States). Cells were harvested 48 h post transfection and assayed for luciferase activities using the Dual Luciferase Reporter Assay Kit (Promega, Madison, WI, United States).

### Statistical Analysis

Chi-square test and Student’s *t*-test were used to compare the distributions of categorical variables and continuous variables between patients and controls. The odd ratios (ORs) and 95% confidence intervals (95% CI) for each genotype were calculated by multivariate logistic regression to determine the association between the selected SNPs and NPC susceptibility. Kaplan-Meier analysis and Cox proportional hazards regression were applied to evaluate the overall survival of NPC patients. The SPSS 19.0 was used for statistical analyses (Chicago, IL, United States) and *P* < 0.05 was set as the threshold of statistically significant.

## Results

### Population Characteristics

The detailed information of the NPC cases and controls were summarized in Supplementary Table 1, as we have previously reported (Guo et al., 2019). There is no distribution difference in the cases and controls for age and BMI (*P* > 0.05). However, the male-to-female ratio was higher in the cases than controls, which was in line with NPC epidemiology. The cases were more likely to have ever smoked (*P* < 0.05) and drunk patients (*P* < 0.05). To eliminate the influence of these factors, the covariant were further adjusted in multivariate logistics regression analysis.

### Linc00312 SNPs Were Associated With NPC Susceptibility

The genotyping call rate of the selected SNPs were greater than 95%, and the genotype frequency of the candidate SNPs were in accordance with Hardy-Weinberg equilibrium, as shown in Table 1. We used multivariate logistic regression analysis to estimate the association between selected SNPs and NPC susceptibility. We found that individuals with rs12497104 GA genotype were associated with significantly increased risk of NPC (GA *vs* GG, OR = 1.437, *P* = 0.003) (Table 1). Individuals with rs15734 GA genotype showed a 22.2% lower risk of NPC than those with rs15734 GG genotype (GA *vs* GG, OR = 0.778, *P* = 0.031). As for rs164966, a 21.9% decreased risk was observed in rs164966 GA genotype carriers in comparison with rs164966 AA genotype carriers (GA *vs* AA, OR = 0.781, *P* = 0.033). Since gender might be a risk factor for NPC, we conducted the stratified analysis to eliminate the influence of gender. As shown in Supplementary Table 2, the ORs of each SNP were even more remarkable in the stratified group than in the whole cohort.

**TABLE 1:** Association between the candidate single nucleotide polymorphisms (SNPs) of linc00312 and nasopharyngeal carcinoma (NPC) susceptibility was analyzed by multivariate logistic regression.

Genotype	Controls *N* (%)	Cases *N* (%)	OR^a^ (95% CI)	*P*^a^
**rs12497104 (G > A)**				
GG	328 (40.1)	217 (35.3)	1.00 (reference)	
GA	356 (43.5)	318 (51.8)	1.437 (1.128–1.830)	**0.003**
AA	134 (16.4)	79 (12.9)	1.012 (0.716–1.430)	0.946
AA + GA *vs* GG			1.325 (1.053–1.667)	**0.016**
AA *vs* GA + GG			0.826 (0.602–1.134)	0.238
**rs15734 (G > A)**				
GG	439 (53.5)	399 (58.4)	1.00 (reference)	
GA	326 (39.8)	248 (36.3)	0.778 (0.620–0.977)	**0.031**
AA	55 (6.7)	36 (5.3)	0.661 (0.415–1.054)	0.082
AA + GA *vs* GG			0.761 (0.612–0.946)	**0.014**
AA *vs* GA + GG			0.723 (0.458–1.143)	0.165
**rs164966 (A > G)**				
AA	430 (52.4)	388 (57.1)	1.00 (reference)	
GA	333 (40.6)	249 (36.7)	0.781 (0.622–0.981)	**0.033**
GG	58 (7.0)	42 (6.2)	0.733 (0.470–1.143)	0.170
GG + GA *vs* AA			0.774 (0.623–0.962)	**0.021**
GG *vs* GA + AA			0.803 (0.520–1.240)	0.322

*^*a*^Adjusted for gender, age, BMI, smoking status, and drinking status. The bold value means *P* < 0.05.*

### Linc00312 rs12497104 AA Genotype Was Correlated With Poorer Overall Survival of NPC

**FIGURE 1:**
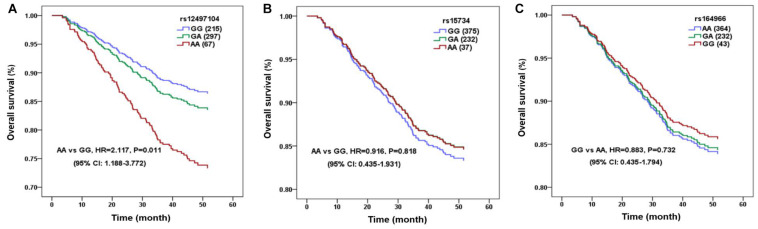
Linc00312 single nucleotide polymorphisms (SNPs) are associated with OS of nasopharyngeal carcinoma (NPC) patients. The survival curve of NPC patients with different genotypes of rs12497104 **(A)**, rs13734 **(B)**, and rs164966 **(C)**.

We included 650 NPC patients in the survival analysis and the detailed therapeutic information was listed in Table 2. The median follow-up duration was 49.27 months (range, 3.5–60 months). The 3-years OS rate of patients with rs12497104 GG genotype were 85.6% and patients with AA genotype were 74.6%. The univariate analysis indicated that the covariates correlated with OS were rs12497104, age, gender, clinical stage, irradiation dose and chemotherapeutic regimen (*P* < 0.05). Multivariate analysis adjusting for these covariates by Cox proportional hazards regression model was used to assess the role of rs12497104. As shown in Figure 1, rs12497104 AA genotype carriers showed a poorer overall survival than GG genotype carriers (AA *vs* GG, HR = 2.117, *P* = 0.011). The mean survival time was 53.56 months for rs12497104 GG genotype carriers and 48.71 months for AA genotype patients. No significant difference in survival was noted in rs15734 and rs164966.

**TABLE 2:** The clinical and treatment information of NPC patients.

Patient characteristics	*N* = 650 (%)	Patient characteristics	*N* = 650 (%)
**Gender**		**T-staging**	
Male	481 (74.0)	T1-T2	312 (48.0)
Female	169 (26.0)	T3-T4	338 (52.0)
**Age, years**		***N*-staging**	
Mean ± SD	48.0 ± 9.7	N0-N1	130 (20.0)
<48	341 (52.5)	N2-N3	520 (80.0)
≥48	309 (47.5)	**M-staging**	
**BMI**		M0	612 (94.2)
<18.5	43 (6.6)	M1	38 (5.8)
18.5 ∼ 24	354 (54.5)	**IC regimen**	
≥24	253 (38.9)	DP	234 (39.2)
**Smoking status**		FP	99 (16.6)
Smoker	313 (48.2)	TP	253 (42.4)
Non-smoker	337 (51.8)	GP	11 (1.8)
**Drinking status**		**CCRT regimen**	
Drinker	122 (18.8)	FP	89 (15.3)
Non-drinker	528 (81.2)	TP	131 (22.5)
**Histological type**		DDP	98 (16.9)
WHO type II	263 (40.5)	NDP	195 (33.6)
WHO type III	287 (59.5)	DP	68 (11.7)
**Clinical stage**		**pGTVnx**	
I-II	73 (11.2)	Mean ± SD	71.1 ± 3.7
III-IV	577 (88.8)	<71.1 Gy	331 (52.7)
		≥71.1 Gy	297 (47.3)

### Genotype-Specific Expression Effect of the Candidate SNPs

**FIGURE 2:**
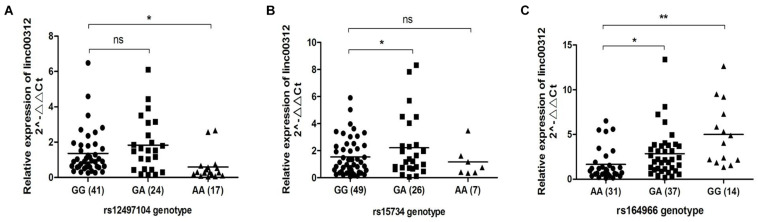
Genotype-specific expression effect of the candidate SNPs. **(A)** The relative expression of linc00312 in different rs12497104 genotype carriers. **(B)** The relative expression of linc00312 in different rs15734 genotype carriers. **(C)** The relative expression of linc00312 in different rs164966 genotype carriers. *P < 0.05; **P < 0.01.

The three candidate SNPs are located in the regulatory region of linc00312 that could cause miRNA–lncRNA binding site gain or loss based on the bioinformatic prediction (Table 3). We speculated the SNPs might regulate the expression of linc00312 by affecting the interaction with miRNAs and we detected the expression level of linc00312 in NPC tissues with different genotypes. The result demonstrated that rs12497104 AA genotype was associated with a significantly lower expression of linc00312 in comparison with rs12497104 GG genotype (*P* < 0.05) (Figure 2A). The expression of linc00312 in rs15734 GA genotype carriers were higher than rs15734 GG genotype carriers (*P* < 0.05) (Figure 2B). Moreover, patients with rs164966 GA and GG genotypes all displayed a higher expression of linc00312 compared with rs164966 AA genotype carriers (*P* < 0.05) (Figure 2C).

**TABLE 3:** Prediction of candidate SNPs causing miRNA-lncRNA gain or loss by TargetScan and miRanda.

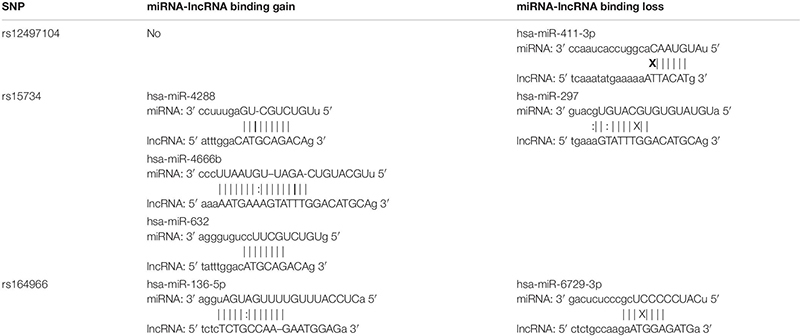

**FIGURE 3:**
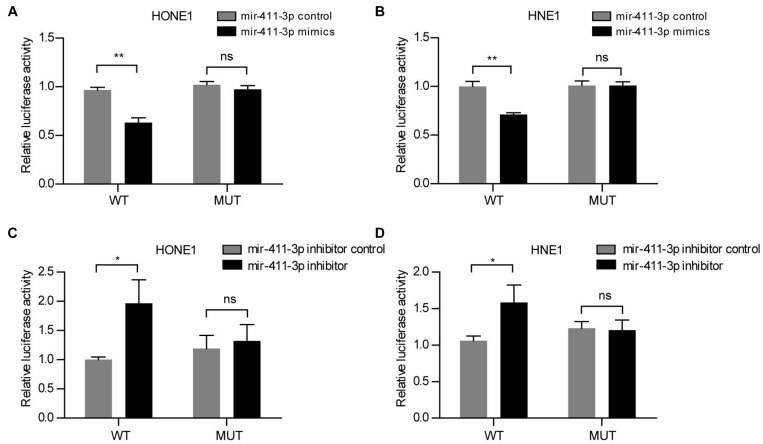
rs12497104 destroys the binding between mir-411-3p and linc00312. **(A)** Relative luciferase activity of the wild type linc00312 (WT) and rs12497104 mutant type linc00312 (MUT) reporter vector that co-transfected with mir-411-3p mimics or mir-411-3p control in HONE1 cells. **(B)** Relative luciferase activity of the WT and MUT reporter vector that co-transfected with mir-411-3p mimics or mir-411-3p control in HNE1 cells. **(C)** Relative luciferase activity of the WT and MUT reporter vector that co-transfected with mir-411-3p inhibitor or mir-411-3p inhibitor control in HONE1 cells. **(D)** Relative luciferase activity of the WT and MUT reporter vector that co-transfected with mir-411-3p inhibitor or mir-411-3p inhibitor control in HNE1 cells. *P < 0.05; **P < 0.01.

Actually, we verified that rs12497104 could destroy the interaction between linc00312 and mir-411-3p by luciferase reporter assay. The relative luciferase activity was significantly decreased when mir-411-3p mimics was co-transfected with the wild-type linc00312 (WT) reporter (*P* < 0.01). On the contrary, the luciferase activity was almost unchanged when mir-411-3p mimics was co-transfected with the rs12497104 mutant linc00312 (MUT) reporter (*P* > 0.05) (Figures 3A,B). What’s more, cotransfection of mir-411-3p inhibitor caused a notable gain on the luciferase activity of the WT reporter (*P* < 0.05) while the luciferase activity of MUT reporter displayed no visible change (*P* > 0.05) (Figures 3C,D).

### *In silico* Prediction of the Candidate SNPs on Linc00312 Folding Structure

**FIGURE 4:**
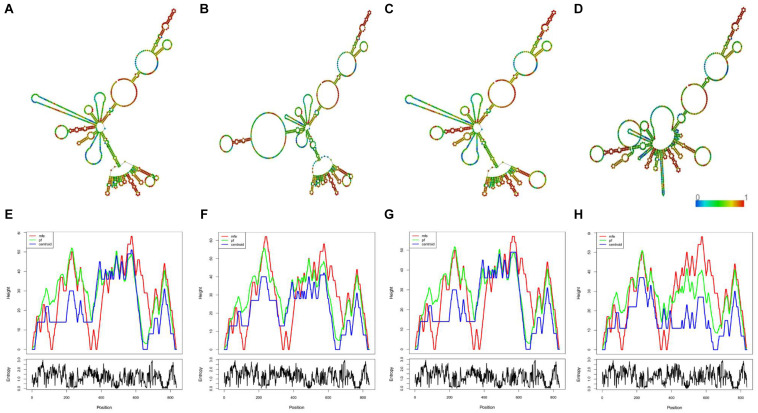
*In silico* prediction of the candidate SNPs on linc00312 folding structure. **(A)** The local centroid secondary structure of the wild type linc00312. **(B)** The local centroid secondary structure of rs12497104 mutant linc00312. **(C)** The local centroid secondary structure of rs15734 mutant linc00312. **(D)** The local centroid secondary structure of rs164966 mutant linc00312. The color of each base represents base-pair probabilities. **(E)** The mountain plot of the wild type linc00312. **(F)** The mountain plot of rs12497104 mutant linc00312. **(G)** The mountain plot of rs15734 mutant linc00312. **(H)** The mountain plot of rs164966 mutant linc00312. The mountain plot presents the MFE structure, the thermodynamic ensemble of RNA structures, the centroid structure and the positional entropy for each position. All of the results were computed by RNAfold WebServer.

By *in silico* analysis, we predicted weather the candidate SNPs could possibly affecting the folding structure of linc00312. As shown in Figure 4, the local centroid secondary structure and minimum free energy of linc00312 were changed following the candidate SNPs alterations. The minimum free energy of the local centroid secondary structure of linc00312 changed from −121.60 to −98.42, −127.20, and −113.50 kcal/mol for rs12497104, rs15734, and rs164966 variations, respectively.

## Discussion

Considering the critical role of linc00312 in NPC, we wondered if the SNPs in linc00312 might serve as potential biomarkers for NPC. In the present study, we identified three SNPs of linc00312 (rs12497104, rs15734, and rs164966) were associated with susceptibility of NPC. In the following survival analysis, we revealed rs12497104 was an independent risk factor for the prognosis of NPC.

Not only for NPC, linc00312 also displayed a vital role in other types of cancers via regulating diverse cellular processes. As for non-small cell lung cancer (NSCLC), patients with lower expression of linc00312 were correlated with larger tumor size, advanced clinical stages as well as shorter overall survival. Overexpression of linc00312 was able to repress cell growth and induce apoptosis of NSCLC cells (Zhu et al., 2017). The other study demonstrated that linc00312 could promote metastasis and angiogenesis in lung cancer via interacting with the transcription factor YBX1 (Peng et al., 2018). Linc00312 also regulated oral fibrogenesis by binding to YBX1 in oral cavity (Yu et al., 2020). Evidence have shown that linc00312 inhibited cell growth and migration of thyroid cancer cells through suppressing the PI3K/Akt and MMP-9 (Min et al., 2018). By targeting miR-197-3p and miR-21, linc00312 functioned as a competing endogenous RNA (ceRNA) and repressed cell migration and invasion of bladder cancer, thyroid cancer and colorectal cancer (Wang et al., 2016; Liu et al., 2017; Li et al., 2018). Linc00312 could induce cell cycle arrest of hepatocellular carcinoma cell and leading to the suppression of proliferation (Wu et al., 2018). In addition, linc00312 participated in drug resistance as it sensitized ovarian cancer cells to cisplatin by activating the Bcl-2/Caspase-3 pathway (Zhang et al., 2018).

A great number of studies have indicated SNPs of lncRNA have great potential as predictive markers for carcinogenesis, prognosis, and drug resistance (Gao and Wei, 2017; Minotti et al., 2018). Our previous findings have suggested NPC patients with lncRNA GAS5 rs2067079 CT genotypes were linked with an obviously increased risk of severe myelosuppression during concurrent radiochemotherapy period compared with CC genotype carriers (Guo et al., 2017). In addition, NPC patients with lncRNA MEG3 rs10132552 CC genotype were vulnerable to suffering chemoradiotherapy induced anemia (Wang et al., 2017). Nevertheless, nothing has been known about linc00312 SNPs and NPC so far.

Unveiling the molecular mechanism of lncRNAs SNPs that contributing to disease risk is quite important for better understanding the pathogenesis of disease. The SNPs may regulate the function of lncRNA through interfering the interaction between lncRNAs and transcription factors, miRNAs or other binding protein partners. Evidence have proven that linc00673 rs11655237 G > A alteration created a binding site for miR-1231, which diminished the function of linc00673 and lead to increased risk of pancreatic cancer (Zheng et al., 2016). The risk variant of rs11672691 in lncRNA PCAT19 suppresses binding of transcription factor NKX3.1 to the promoter of PCAT19-short, resulting in PCAT19-long activation and prostate cancer growth and metastasis (Hua et al., 2018). Another prostate cancer-associated variant at lncRNA PCAT1 rs7463708 increases binding of transcription factor ONECUT2 to the PCAT1 promoter, resulting in upregulation of PCAT1 and prostate transformation (Guo et al., 2016). In colon cancer, by interacting with the CFIm complex with allele specific affinities, lncRNA CCAT2 rs6983267 regulates the alternative splicing of glutaminase, resulting in reprogramming of cancer metabolism (Redis et al., 2016). To data, literature that clearly clarify the molecular mechanism of cancer related SNPs in lncRNA is very scarce.

Bioinformatics approaches have usually been applied to predict the potential role of SNPs in lncRNAs. In the present study, we applied LncRNASNP, ENSEMBL, and RNAfold for SNP selection and function prediction (Gruber et al., 2008; Miao et al., 2018). Actually, bioinformatics approach is a cost-effective way to screening causal SNPs. The prediction result indicated all of the three SNPs of linc00312 (rs12497104, rs15734, and rs164966) had an eQTL trait and were likely to create or destroy the binding sites of miRNAs with linc00312. In light of this, we wondered if the SNPs could affect the function/expression of linc00312. It has been reported that 75% of the lncRNA SNPs affected the expression level of lncRNA (Kumar et al., 2013). So, we examined the expression of linc00312 with different genotypes and found the genotype specific expression feature. The rs12497104 AA genotype carriers had a lower expression of linc00312 and were subjected to enhanced risk of NPC and poorer survival. Likewise, the patients with GA genotype of rs15734 and rs164966 showed a lower expression of linc00312 and were correlated with decreased risk of NPC. In addition, the local secondary structure and minimum free energy of linc00312 was affected by the SNPs.

Increasing evidences have indicated that lncRNA could function as ceRNA to regulate mRNA by sponging miRNA. For the first time, we proved that rs12497104 could interfere the binding between linc00312 and mir-411-3p. This finding provided one probable mechanism by which rs12497104 affected the function of linc00312. As far as we know, there was only two miRNAs (mir-197-3p, mir-21) have been identified to interact with linc00312 (Wang et al., 2016; Liu et al., 2017; Li et al., 2018). To date, the biological function of mir-411-3p is still largely unknown. A recent study reported mir-411-3p interacted with lncRNA ANRIL and inhibited the malignant proliferation and tumor stem cell like property of multiple myeloma (Wang et al., 2020). Another study demonstrated lncRNA TTN-AS1 could function as the mir-411-3p sponge in oral squamous cell carcinoma (OSCC) and mir-411-3p exerted the inhibitory functions on OSCC growth (Fu et al., 2020). In addition, lncRNA CDKN2B-AS1 could interact with mir-411-3p and contribute to carcinogenesis in ovarian cancer (Wang et al., 2019). The role of mir-411-3p in NPC still needs further investigation.

In conclusion, we demonstrated the SNPs of linc00312 were associated with NPC susceptibility and survival possibly by influencing the expression of linc00312. Our findings may shed some light on the biomarkers for predicting NPC risk and prognosis.

## Data Availability Statement

The original contributions presented in the study are included in the article/Supplementary Material, further inquiries can be directed to the corresponding authors.

## Ethics Statement

The studies involving human participants were reviewed and approved by the Independent Ethical Committee of Institute of Clinical Pharmacology, Central South University. The patients/participants provided their written informed consent to participate in this study.

## Author Contributions

ZG performed the experiments, analyzed the data, and drafted the manuscript. M-HB helped for sample collection. Y-XF, YZ, and H-YL performed the quality control of data. X-LZ and BW provided the technical assistance. B-SH and Q-QL participated in manuscript editing. J-YL participated in manuscript review. All authors read and approved the final manuscript.

## Conflict of Interest

Q-QL was employed by Genesis Beijing Co., Ltd. The remaining authors declare that the research was conducted in the absence of any commercial or financial relationships that could be construed as a potential conflict of interest.
