# Supplementary material for: Genetic Polymorphisms of Long Non-coding RNA Linc00312 Are Associated With Susceptibility and Predict Poor Survival of Nasopharyngeal Carcinoma
Source: Front Cell Dev Biol. 2021 Jul 16;9:698558. doi: 10.3389/fcell.2021.698558 (PMC8322760; doi:10.3389/fcell.2021.698558)
Supplement: Supplementary file 1 [file Table_1.DOCX]

**Supplementary Table 1. The basic information of the NPC cases and cancer-free controls.**

| **Variables** | **Controls N (%)** | **Cases N (%)** | ***P ^a^*** |
| --- | --- | --- | --- |
| **Gender** |  |  |  |
| Male | 378 (45.9) | 517 (75.6) | **<0.001** |
| Female | 445 (54.1) | 167 (24.4) |  |
| **Age, years** |  |  |  |
| ≥ 47 | 349 (42.4) | 305 (44.6) | 0.394 |
| < 47 | 474 (57.6) | 379 (55.4) |  |
| **BMI** |  |  |  |
| ≥ 24 | 312 (37.9) | 275 (40.2) | 0.542 |
| 18.5 ~ 24 | 466 (56.6) | 368 (53.8) |  |
| ≤ 18.5 | 45 (5.5) | 41 (6.0) |  |
| **Smoking status** |  |  |  |
| Smoker | 192 (23.3) | 326 (47.7) | **<0.001** |
| Nonsmoker | 631 (76.7) | 358 (52.3) |  |
| **Drinking status** |  |  |  |
| Drinker | 121 (14.7) | 128 (18.7) | **0.043** |
| Nondrinker | 702 (85.3) | 556 (81.3) |  |

Note: People who never smoked were defined as nonsmokers and others were defined as smokers. People who never drunk were defined as nondrinkers and others were defined as drinkers.

^a^ Two-sided χ^2^ test.

**Supplementary Table 2. Stratified analysis of the candidate SNPs and NPC susceptibility by gender.**

|  | **Genotype** | **Controls N (%)** | **Cases N (%)** | **OR^a^(95% CI)** | ***P ^a^*** |
| --- | --- | --- | --- | --- | --- |
| **male** | **rs12497104 (G>A)** |  |  |  |  |
|  | GG | 163 (43.1) | 168 (32.5) | 1.00 (reference) |  |
|  | GA | 160 (42.3) | 237 (45.8) | 1.463 (1.085-1.972) | **0.013** |
|  | AA | 52 (13.8) | 57 (11.0) | 1.087 (0.700-1.688) | 0.711 |
|  | **rs15734 (G>A)** |  |  |  |  |
|  | GG | 194 (51.3) | 290 (56.1) | 1.00 (reference) |  |
|  | GA | 152 (40.2) | 200 (38.7) | 0.866 (0.652-1.151) | 0.321 |
|  | AA | 31 (8.2) | 26 (5.0) | 0.558 (0.318-0.980) | **0.042** |
|  | **rs164966 (A>G)** |  |  |  |  |
|  | AA | 193 (51.1) | 283 (54.7) | 1.00 (reference) |  |
|  | GA | 151 (39.9) | 200 (38.7) | 0.887 (0.666-1.179) | 0.408 |
|  | GG | 33 (8.7) | 31 (6.0) | 0.639 (0.374-1.089) | 0.100 |
|  |  |  |  |  |  |
| **female** | **rs12497104 (G>A)** |  |  |  |  |
|  | GG | 165 (37.1) | 49 (29.3) | 1.00 (reference) |  |
|  | GA | 196 (44.0) | 81 (48.5) | 1.386 (0.916-2.096) | 0.123 |
|  | AA | 82 (18.4) | 22 (13.2) | 0.880 (0.494-1.568) | 0.665 |
|  | **rs15734 (G>A)** |  |  |  |  |
|  | GG | 245 (55.1) | 109 (65.3) | 1.00 (reference) |  |
|  | GA | 174 (39.1) | 48 (28.7) | 0.605 (0.407-0.900) | **0.013** |
|  | AA | 24 (5.4) | 10 (6.0) | 0.954 (0.441-2.064) | 0.905 |
|  | **rs164966 (A>G)** |  |  |  |  |
|  | AA | 237 (53.3) | 105 (62.9) | 1.00 (reference) |  |
|  | GA | 182 (40.9) | 49 (29.3) | 0.594 (0.400-0.882) | **0.010** |
|  | GG | 25 (5.6) | 11 (6.6) | 1.012 (0.480-2.135) | 0.974 |

^a^ Adjusted for age, BMI, smoking status and drinking status.
